# The Scent of Spoils

**Published:** 1896-12

**Authors:** 


### THE SCENT OF SPOILS.

Already the searchers after place are entering the field and scenting the fruits of victory, are waylaying their friends and pleading with the bosses and chief spoils-politicians for their favor and influence. These same bosses and place-givers they have time and again condemned personally and professionally, and it is the same system from which they have longed to be freed. The same liberty which is accorded other men in professional places, and which they have so often poured forth their lamentations to have, they are now willing to forego if only the favor of place may fall to them, and will tear their hair with rage if they are disappointed. Fellow-veterinarians, if this merit system, now so thoroughly engrafted on our political system, and which is of such great importance to the veterinary profession, is a good thing, why will you endanger it by these importunities? Why will you adopt those methods of securing recognition by which are created these obnoxious bosses, who, in rendering you this little service, are enabled to inflict the most serious injuries upon the whole people by vicious legislation, which is always the outcome of corrupt use of political power? Let every veterinarian do his duty and strengthen the hands of the incoming administration, that it may extend its beneficent influences in the advancement of the merit system, by which veterinary service will be raised, its value to our Government enhanced, the compensation made more adequate, and the need of an increased service made more evident, and thus every honest line of our profession advanced. Never was a more pernicious system destroyed; never a more disgraceful method of filling public place eliminated. Surely we have all suffered enough from its baneful results, and should henceforth lift up our voices and our influence against the rehabilitation of the infamous spoils system. No temporary abatement of its wise provisions should be tolerated by the incoming administration without bringing down upon itself the most scornful reproach of every good citizen and of every true veterinarian. SUSTAIN THE PRESENT SYSTEM!

